# Emerging technologies and infection models in cellular microbiology

**DOI:** 10.1038/s41467-021-26641-w

**Published:** 2021-11-19

**Authors:** Ana Teresa López-Jiménez, Serge Mostowy

**Affiliations:** Department of Infection Biology, London School of Hygiene and Tropical Medicine, Keppel Street, London, WC1E 7HT UK

**Keywords:** Infectious diseases, Infection, Biological techniques, Cellular microbiology, Mechanisms of disease

## Abstract

The field of cellular microbiology, rooted in the co-evolution of microbes and their hosts, studies intracellular pathogens and their manipulation of host cell machinery. In this review, we highlight emerging technologies and infection models that recently promoted opportunities in cellular microbiology. We overview the explosion of microscopy techniques and how they reveal unprecedented detail at the host-pathogen interface. We discuss the incorporation of robotics and artificial intelligence to image-based screening modalities, biochemical mapping approaches, as well as dual RNA-sequencing techniques. Finally, we describe chips, organoids and animal models used to dissect biophysical and in vivo aspects of the infection process. As our knowledge of the infected cell improves, cellular microbiology holds great promise for development of anti-infective strategies with translational applications in human health.

## Abstract (web summary)

Cellular microbiology is a field that combines the study of infection and cell biology. In this review, we highlight emerging technologies and infection models that recently transformed our understanding of the infected cell and may inspire future medicine.

## Introduction

Over the past three decades, pioneering scientists including Stanley Falkow, Pascale Cossart and Philippe Sansonetti innovated the study of cellular microbiology to discover molecular mechanisms by which microbes infect their host and cause disease. These studies enabled landmark discoveries in bacterial pathogenesis and host cell biology. The investigation of cytoskeleton rearrangements induced by bacterial pathogens, including *Listeria* and *Shigella*, are central to the field of cellular microbiology (reviewed in Stradal and Schelhaas^1^) and one of the best examples of infection biology contributing to fundamental cellular processes comes from the reconstitution of bacterial ‘*actin tails*’ (Box 1) in vitro^2^. In this review, we focus on bacterial infection and describe how the field of cellular microbiology has recently been transformed by emerging technologies and infection models, highlighting studies that revolutionised our understanding of pathogenesis and fundamental cellular processes.

Box 1 Glossary of key terms **Actin tails**: filamentous actin structures polymerised by some intracellular bacterial pathogens to propel their movement in the host cell cytosol for dissemination.

**Autophagy**: catabolic pathway for the degradation of cytosolic material enclosed in a double membrane vesicle (called autophagosome).

**Bacterium-containing vacuole (BCV)**: intracellular vesicular compartment that results from the internalisation of bacteria into host cells.

**Cell-autonomous immunity**: ability of immune and non-immune cells to protect themselves against intracellular bacterial pathogens using highly conserved defence pathways (including autophagy and the production of antimicrobial peptides).

**Click chemistry**: bioorthogonal reactions in which small biocompatible reporters are conjugated to biomolecules of interest, used in various live-cell applications (including photoactivable fluorogenic labelling of proteins and lipids).

**CRISPR libraries**: collection of single guide RNA (sgRNA) molecules for subsequent CRISPR/Cas-9 gene editing (CRISPR knockout) or transcriptional regulation (CRISPR activation, interference).

**Deubiquitinase**: protease that cleaves ubiquitin from ubiquitinated proteins for their localisation, activity and dynamics.

**Diffraction limit**: minimum angular separation between two objects that can be distinguished by an optical microscope.

**Galectins**: type of lectins or carbohydrate-binding proteins with affinity for β-galactosides, used as a marker for membrane damage at the BCV as it binds to luminal glycans when exposed to the cytosol.

**Guanylate-binding proteins (GBPs)**: family of IFN-inducible GTPases that target host membranes and bacterial surfaces to regulate inflammasome activity and antibacterial defence mechanisms.

**High-content screenings**: phenotypic screenings focused on the complex analysis of multiple parameters (such as number, morphology and dispersion of intracellular bacteria, in combination with the recruitment of host defence markers upon silencing of different host genes).

**High-throughput screenings**: phenotypic screening focused on the rapid analysis of multiple candidates, normally based on a single parameter (such as the growth of intracellular bacteria during multiple drug treatments).

**Inflammasome**: multiprotein complex that mediates secretion of pro-inflammatory cytokines and programmed cell death.

**Macropinosomes**: vesicles produced as a result of macropinocytosis, a type of endocytosis that involves non-specific uptake of extracellular material.

**M cells**: specialised epithelial cells in the intestine important for host defence.

**Microfluidic chips**: device containing micro-chambers, channels and inlet/outlet ports that enable analysis of single cells by microscopy.

**Neutrophil extracellular traps (NETs)**: networks (composed of DNA and proteins) with antimicrobial activity that are secreted by neutrophils in response to extracellular pathogens.

**Nuclear Factor-κB (NF-κB)**: protein complex that regulates transcription of immune factors and pro-inflammatory molecules (such as cytokines) in response to infection.

**NOD-like receptors (NLRs)**: highly conserved pattern recognition receptors (PRRs) that recognise cytosolic danger signals for cell-autonomous immunity.

**Organoids**: self-organised 3D tissues derived from stem or progenitor cells, which resemble a specific organ.

**Peyer’s patches**: specialised lymphatic tissue present in the small intestine organised as follicles.

**Phenotypic heterogeneity**: in the case of bacterial cells, the presence of different phenotypes (e.g. susceptibility to antibiotic treatment) that naturally occurs within an isogenic population.

**Post-translational modifications**: covalent modification of proteins that enables their modulation (regulating protein localisation, activity, dynamics) in response to environmental stimuli.

**Septins**: group of GTP-binding cytoskeletal proteins that assemble to form filaments, bundles, rings and cage-like structures.

**Tolerance**: host mechanism during infection that limits pathology (but does not restrict the pathogen) and promotes survival.

**Type III Secretion System (T3SS)**: secretion machinery found in Gram-negative bacteria, essential for invasion and pathogenicity of enteric bacteria (such as *S*. Typhimurium and *S. flexneri*).

**Type VI Secretion System (T6SS)**: secretion machinery found in Gram-negative bacteria (such as *V. cholerae* and *P. aeruginosa*), essential for mediating bacterial interactions during competition.

**Type VII Secretion System (T7SS)**: secretion machinery found in Gram-positive bacteria (such as *L. monocytogenes* and *S. aureus*) and mycobacteria (such as *M. tuberculosis*), playing important roles in bacterial physiology, competition and virulence.

**Ubiquitination**: post-translational modification in which a protein or lipid is covalently linked with one or more ubiquitin residues, in a three step enzymatic process involving a ubiquitin-activating enzyme (E1), ubiquitin-conjugating enzyme (E2) and ubiquitin-ligase (E3).

## Cellular microbiology imaged by state-of-the-art microscopy

**Fig. 1:**
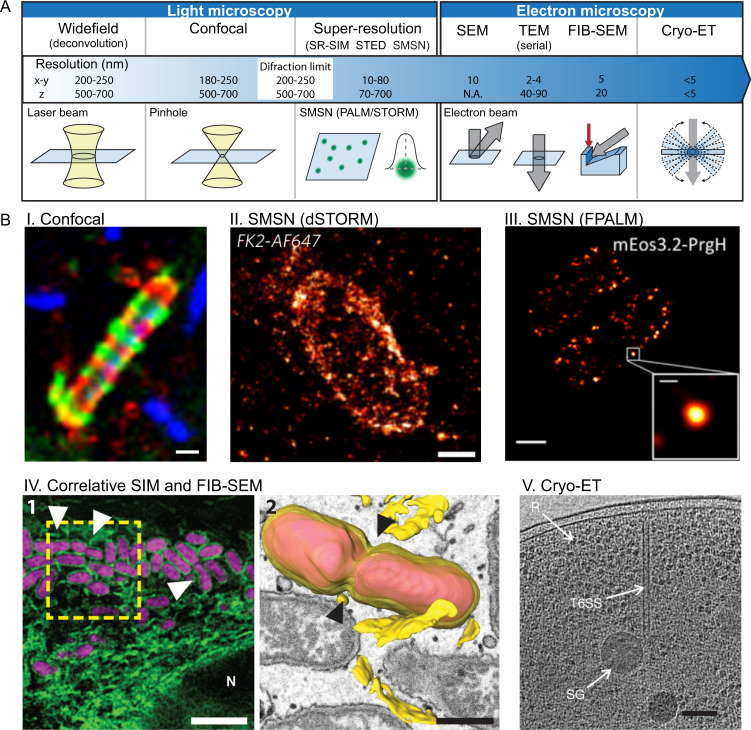
State-of-the-art microscopy techniques recently used to visualise the infection process. **A** Scheme of selected microscopy techniques used for cellular microbiology, including diagrams and resolution. More detailed information on the x, y and z resolution of the different microscopy techniques is reviewed in Schermelleh et al.^3^. **B** I, Septin cage entrapment of cytosolic *S. flexneri* imaged with confocal microscopy, scale bar = 1 μm (figure adapted from Mostowy et al.^95^). II, Cytosolic *S*. Typhimurium decorated with ubiquitin visualised with dSTORM, scale bar = 1 μm (figure adapted from Van Wijk et al.^12^). III, T3SS of *S*. Typhimurium observed by FPALM, scale bar = 0.5 μm (figure adapted from Zhang et al.^7^). IV, *B. abortus* infection of HeLa cells expressing the ER marker Emerald–Sec61β (green) observed by correlative SIM (1, scale bar = 3 μm) and FIB-SEM with 3D reconstitution of the rBCV membrane and adjacent ER cisternae (2, scale bar = 500 nm μm, figure adapted from Sedzicki et al.^26^). V, T6SS structure of *V. cholerae* imaged with Cryo-ET (figure adapted from Basler et al.^96^). R labels putative ribosomes and SG labels a polyphosphate storage granule, scale bar = 100 nm. SR super-resolution, SIM structured illumination microscopy, STED stimulated emission depletion microscopy, SMSN single-molecule switching nanoscopy.

Standard widefield and confocal microscopy techniques historically provided the scale and resolution required to enable major discoveries in cellular microbiology, such as mechanisms underlying the internalisation and actin-based motility of invasive bacterial pathogens. In this section, we highlight state-of-the-art microscopy techniques providing unprecedented opportunities to investigate bacteria–host cell interactions (Fig. 1 and Table 1A).

**Table 1:** Advantages, disadvantages and future directions for cellular microbiology techniques.

	Advantages	Disadvantages	Future directions
**A. Microscopy**			
Widefield	• Convenient for live imaging, thick optical sections	• Resolution not ideal for subcellular structures	• Imaging through different scales: from the single bacterial cell to the whole animal level • Imaging emerging and neglected pathogenic microbes as well as clinically relevant strains
Confocal	• Commercial set-ups are widely available	• Slow speed and high laser intensity are inconvenient for live samples (except spinning disc)	Same as above
Super-resolution	• Revolutionary for subcellular imaging and single-molecule quantification	• Custom-built set-ups and complex image analysis pipelines are common	Same as above
SEM, TEM, FIB-SEM	• Enables precise localisation of proteins in combination with fluorescence microscopy (correlative microscopy)	• Requires chemical fixation and dehydration; during FIB-SEM sample is destroyed with successive milling	• Increased availability and accessibility Closing the gap between structural detail and functional relevance
**B. Discovering targets**			
High-throughput/content screenings	• Process can be fully automated Datasets can be reanalysed to test a new hypothesis	• Trade-off between the speed of acquisition and resolution Challenging data analysis, management and storage	• Flexible acquisition with integrated and iterative analysis Screening in organoids or optically accessible animal models (e.g. zebrafish larvae)
Biochemical mapping	• In situ biotinylation High temporal and spatial resolution	• High false positive rate Lengthy tag can impair protein localisation and function	• Novel bioengineered enzymes with higher efficiency and faster labelling times Simultaneous mapping of multiple POIs
Dual RNA-sequencing	• Simultaneous host and pathogen transcriptome Detection of regulatory RNAs	• Low yield of isolated RNA mRNA and protein abundance may not correlate	• Single-cell dual RNA-seq, spatial transcriptomics RNA-seq of multiple bacteria species inside the host (e.g. microbiota)
**C. Cellular microbiology infection models**			
Chips	• Studies at the single-cell level, relevant to dissect phenotypic heterogeneity Useful to measure the impact of drugs on bacterial replication, morphology and infection dynamics	• Dedicated platforms limited to selected bacterial species or specific host cell lines Specialised equipment, protocols and image analysis tools not yet widely accessible	• Microfabrication facilities and procedures more standardised and easily accessible Increased versatility, useful for multiple cell lines and pathogens
Organ-on-chips	• Approaching organ complexity and different cell types (e.g. epithelial cells, macrophages) can be sequentially added Useful to investigate physical parameters and mechanical forces that impact infection	• Limited number of organ-on-chip platforms developed so far Organ-on-chip design limits the parameters to study	• More faithful recapitulation of multi-tissue organs Increased ability to Influence physical parameters of the infection/colonisation process (e.g. flow, movement)
Organoids	• Closely resembles organ physiology (brain, lung, intestine) and reduces the number of animals used in research Useful to model genetic determinants underlying human disease (when derived from mice or patients carrying mutations)	• Limited diversity of organs developed so far Difficult to scale-up production for high-throughput studies	• Organoids derived from patient primary cells Studies using complex bacterial communities (e.g. microbiota)

(A) State-of-the-art microscopy techniques (highlighted in Fig. 1). (B) Approaches used to discover targets important during infection (highlighted in Fig. 2). (C) Innovative platforms used to dissect the infection process (highlighted in Fig. 3).

### Light microscopy techniques

An important breakthrough in the field of fluorescence microscopy was the invention of super-resolution microscopy (reviewed in Schermelleh et al.^3^). Super-resolution microscopy comprises an expanding family of techniques with a common characteristic: their ability to surpass the ‘diffraction limit’. These include structured illumination microscopy (SIM), stimulated emission depletion microscopy (STED) and single-molecule switching nanoscopy (SMSN), which have been developed concomitantly with improved dyes, probes and labelling methods^4^. Super-resolution microscopy is instrumental to focus on individual molecules and access previously unresolvable subcellular structures, such as the bacterial cytoskeleton, cell wall and nucleoid, as well as the highly structured and compartimentalised bacterial cytoplasm (reviewed in Singh and Kenney^5^). SMSN methods are based on photoswitchable molecules, so that their emission signals are spatiotemporally separated and signal centres can be determined with nm resolution. As a consequence, SMSN can reveal detailed insights into the architecture, stoichiometry and localisation of subcellular structures^6^. While originally these techniques had limited axial resolution (2D SMSN), the use of dual detectors with ‘4Pi geometry’ increased the localisation precision in z (3D or 4Pi-SMSN)^6^. Both 2D and 3D SMSN were used to investigate the ‘Type III Secretion System (T3SS)’ of *Salmonella enterica* serovar Typhimurium^7^, an important zoonotic pathogen causing gastroenteritis and inflammation of the intestinal mucosa. Although the T3SS needle complex has been isolated and studied in depth, the organisation of the entire T3SS (including the sorting platform) was less understood. In a recent study using SMSN, authors revealed the ultrastructure, protein stoichiometry, assembly and distribution of the T3SS in live bacterial cells, as well as the localisation of bacterial effectors prior to secretion^7^. Super-resolution microscopy offered fundamental insights into the secretion of bacterial effectors and growth of bacteria in broth, and in the future can be used to study bacterial pathogens inside cells. However, this remains challenging, considering that super-resolution microscopy is sometimes laborious (e.g. dye calibration) and often requires high laser intensities incompatible with living samples. The continuous improvements to super-resolution microscopy (wider accessibility, intuitive user interface, better optics and standardised analysis) will increase the number of studies exploring single-cell bacteria physiology during infection and reveal further adaptations to different intracellular niches.

While *Salmonella* is viewed as an intracellular pathogen that resides in a ‘bacterium-containing vacuole (BCV)’, a small percentage of bacteria rupture the BCV and escape to the cytosol where they hyper-replicate. However, to prevent hyper-replication, the luminal leaflet of the broken BCV is recognised by ‘galectins’^8^, and the LPS of cytosolic bacteria become ‘ubiquitinated’^9,10^. These events recruit selective autophagy cargo receptors, including p62/SQSTM1, NDP52/CALCOCO2 and OPTN, that target *Salmonella* to degradation by autophagy. While standard confocal microscopy provided evidence that autophagy cargo receptors arrange in distinct microdomains at the surface of *Salmonella*^11^, the precise arrangement of ubiquitin on the surface of *S*. Typhimurium was uncovered by direct Stochastic Optical Reconstruction Microscopy (dSTORM)^12^. In this study, the ‘deubiquitinase’ OTULIN was shown to restrict bacterial growth by controlling the density of linear ubiquitin chains on the bacterial surface, which serves as a platform for NF-κB activation.

Finally, lattice light-sheet microscopy (LLSM) is a newly developed super-resolution microscopy method, which capitalises on previous microscopy techniques (light-sheet microscopy, SIM) and provides gentle and fast super-resolution in a volumetric sample^13^. Here, multiple thin sheets of light illuminate the specimen (whose scale comprises several orders of magnitude: from single cells to whole animals) providing fast imaging with high axial resolution and minimal photobleaching. The application of LLSM has been limited by the complexity of set-ups being custom-built, and also by the requirement of computer-intensive postprocessing procedures. Consistent with this, LLSM has not yet been used to study infection. However, mitochondria can be viewed as ancient bacterial cells, and a study using LLSM showed that mitochondria make actin tails similar to those of *Rickettsia* (an obligate intracellular pathogen and α-proteobacteria closely-related to mitochondria)^14^. The recent release of commercial LLSM will undoubtably expand its use in infection biology.

In the absence of sophisticated optics, a resolution higher than offered by conventional fluorescence microscopy can be achieved using expansion microscopy. Here, the sample is cross-linked to a polyelectrolyte hydrogel which has the capacity to swell when immersed in water. This enables physical expansion of the biological sample (up to 100 times), whose molecules separate in an isotropic manner^15^. Expansion microscopy can be used on its own, or its resolution can be enhanced when used in combination with conventional super-resolution microscopy techniques as demonstrated by the study of eukaryotic structures^16^. Its application to bacteria has great potential to provide nm-scale insights but it cannot be applied to live samples, and careful precautions are required to ensure that expansion does not affect the localisation or ultrastructure of complexes of interest. Although the expansion of bacteria has been technically challenging considering the rigidity of prokaryotic cell walls, digestion of the cell wall with enzymes (such as lysozyme) has been used^17^. In this study, the authors detected bacterial cell wall damage in untreated *S*. Typhimurium (as cells expanded) during infection of mouse RAW264.7 macrophages. Interestingly, the damage did not occur in all intracellular bacteria homogenously and varied for different bacteria inside the same macrophage. This lack of uniformity in an isogenic population, called ‘phenotypic heterogeneity’, remains a major challenge for effective antibiotic treatment^18^. Recently, a functionalized ceramide has been developed to simultaneously enable fluorescence labelling with Dibenzocyclooctyne (DBCO)-containing dyes (via ‘click chemistry’)^19^ and chemical fixation (with the addition of a primary amine), thus enabling visualisation of lipids during expansion microscopy^20^. This modified ceramide can integrate into membranes of pathogenic bacteria known to exploit host sphingolipids, including *Neisseria gonorrhoeae* and *Chlamydia trachomatis*. In this study, the combined use of click-labelled fluorescent ceramide, expansion microscopy and SIM enabled, for the first time using light microscopy, visualisation of the separated inner and outer membrane of Gram-negative bacteria^20^.

### Electron microscopy techniques

For many decades, transmission and scanning electron microscopy techniques (TEM and SEM, respectively) enabled ultrastructural inspection of infected samples. However, a general limitation of these techniques is the acquisition of 3D information, which can be laborious (i.e. serial section tomography). This limitation was overcome ~10 years ago with the innovation of focused ion beam scanning electron microscopy (FIB-SEM). FIB-SEM combines the serial layered ion milling of a volumetric sample with the orthogonal acquisition of exposed surfaces, to obtain a spatial resolution of ~20 nm in z. For this reason, FIB-SEM is optimal to analyse the 3D morphology of organelles and their association with subcellular structures. Shortly after its innovation, FIB-SEM was applied to investigate the manipulation of host cell organelles by various intracellular pathogens (reviewed in Weiner and Enninga^21^), including BCV membrane rupture caused by several species of mycobacteria^22,23^, which enables bacterial escape to the cytosol and is dependent on the mycobacterial ESX-1 ‘Type VII Secretion System (T7SS)’. Similar to TEM, the full potential of FIB-SEM can be achieved in combination with correlative light and electron microscopy (CLEM), in which ultrastructural information (achieved by electron microscopy) is combined with molecular specificity and positional information provided by a fluorescent marker (visualised by light microscopy). Correlative FIB-SEM has been used to visualise the invasion of *Shigella flexneri* in HeLa cells. Here, the *S. flexneri* BCV was found to be closely surrounded by Rab11 positive ‘macropinosomes’, recruited via bacterial effectors IpgD, IpgB1 and IpgE, which determined the timing of BCV rupture^24^. A separate study showed that *S. flexneri ipgD* mutants are enclosed in a dense actin structure, called a cocoon, proposed to delay bacterial escape to the cytosol^25^. *Brucella* is a vacuolar pathogen that resides in a compartment, which first undergoes phagosomal maturation (endosomal BCV) and then acquires endoplasmic reticulum (ER) characteristics to support bacterial replication (replicative BCV)^26^. Correlative SIM and FIB-SEM revealed that membranes of replicative BCVs form a complex and continuous network with ER cisternae and nuclear membrane, and also showed fusion events between different BCVs^26^. In addition to long acquisition times (several days for a single serial block), one limitation of correlative FIB-SEM studies is that organelle segmentation is often performed manually, which is time-consuming and data tends to be mostly descriptive. Significant efforts are being advanced in the field of computer vision and artificial intelligence for automated segmentation of 3D electron microscopy images^27^, which will reveal architectural features of the infected cell and provide a quantitative aspect to these studies. Another major drawback of electron microscopy techniques such as TEM, SEM or FIB-SEM, is that the preparation of biological samples requires the chemical fixation, dehydration and embedding of the specimen in resin, which can lead to severe artifacts. However, these limitations were overcome with the invention of cryo-electron tomography (cryo-ET). In this case, the sample is rapidly plunged into liquid ethane at ~80 K and becomes vitrified in its hydrated native state. Cryo-ET allows visualisation of any biological structure in its cellular context with a resolution of <5 nm. Although any bacterial structure is potentially amenable to this technique, cryo-ET has been instrumental to capture ultrastructure of different bacterial secretion systems including the Type II Secretion System (T2SS) and Dot/Icm Type IV Secretion System (T4SS) of *Legionella pneumophila,* as well as the ‘Type VI Secretion System (T6SS)’ of *Vibrio cholerae* (reviewed in Oikonomou and Jensen^28^). This information has enabled their structural and evolutionary comparison, providing a detailed understanding of their composition, integration in the bacterial membrane and mechanism of action. Cryo-ET has also been used to explore the precise arrangement of host cell actin during the invasion of *S*. Typhimurium in HeLa cells, which discovered different invasion mechanisms dependent on the *Salmonella* effector SipA^29^. Correlative light and cryo-ET has been exploited to visualise the precise arrangement of ‘septins’ surrounding *S. flexneri* and *Mycobacterium smegmatis*, which (as observed in vitro) assemble at the bacterial cell pole as filaments (and not as bundles, rings, lattices or gauzes) to entrap bacteria in cage-like structures^30^.

Nanoscale secondary ion mass spectrometry (NanoSIMS) is a spectroscopy approach to interrogate the chemical composition of prokaryotic and eukaryotic cells. Here, a focused ion beam is used to scan the sample surface and sputtered secondary ions are analysed by mass spectrometry^31^, which creates a highly sensitive chemical image with nm resolution. NanoSIMS is widely used to study metabolic activities at the single-cell level in microbial ecosystems and has also been applied to study lipid composition, growth dynamics and phenotypic heterogeneity of bacterial cultures. Similar to other types of correlative microscopy, the chemical map obtained with NanoSIMS can be combined with structural information provided by fluorescence and electron microscopy, revealing the chemical composition of cellular organelles. In the case of infection biology, NanoSIMS was first used to determine the subcellular reservoir of antimicrobial drugs in human monocyte-derived macrophages infected with *M. tuberculosis* (the causative agent of human TB)^32^. Here, the authors could trace the presence of bedaquiline (a lipophilic antitubercular compound) because of its bromine atom. Bedaquiline was shown to accumulate in macrophage lipid droplets, where it is progressively transferred to *M. tuberculosis* during infection. The discovery that lipid droplets can serve as a reservoir for lipophilic antibiotics, which impacts the rate and duration of exposure of intracellular bacteria to these drugs, highlights the importance of in vivo pharmacokinetics in the evaluation and administration of antibiotics to combat human infection.

## Discovering targets during bacterial infection

In addition to searching for antibiotic and anti-infectious compounds, screens have provided in depth understanding of the bacterial infection process (Fig. 2 and Table 1B). In 2005, two studies pioneered genome-wide high-throughput microscopy screens to identify host factors involved in bacterial infection^33,34^. In both cases, S2 cells (derived from a *Drosophila* macrophage-like lineage) were treated with a genome-wide siRNA library and subsequently infected with fluorescently labelled *Listeria monocytogenes or Mycobacterium fortuitum* (an opportunistic environmental pathogen) to assess intracellular growth. Since these seminal studies, ‘high-throughput’ and ‘high-content screening’ have been accelerated by the emergence of genome-wide ‘CRISPR libraries’, and technological developments associated with automated microscopy which includes advanced acquisition hardware (such as reliable autofocus, water immersion objectives, robotisation and coupled cell incubators) and improved image processing and analysis tools. Considering the vast amount of data generated, automated pipelines are paramount for fast, unbiased and accurate quantifications. While commercial automated cell imagers are often coupled to dedicated analysis software, many open-access options have become available in the last decade. Some of these perform machine-learning-based classification of phenotypes (CellProfiler Analyst^35^) or segmentation of precise structures (ilastik^36^, Cellpose^37^). Recently, machine-learning-based image analysis platforms (e.g. HRMAn^38^) have been developed for the dedicated analysis of host–pathogen interactions. When applied to *Salmonella* infection of HeLa cells, the percentage of bacteria decorated with ubiquitin were quantified in an automated manner^38^. To date, high-throughput and high-content screenings have been performed for different bacterial infections including *L. pneumophila, C. trachomatis* and *M. marinum* (reviewed in Brodin and Christophe^39^). Quantitative studies are focused on bacterial morphology using bacteria in broth culture. In this case, essential genes of *B. subtilis* were knocked down using CRISPR, and multiple morphological parameters were analysed using an automated single-cell analysis workflow^40^. The authors categorised a wide range of bacterial cell phenotypes based on their morphology and linked phenotypes to gene networks. It will be important to perform similar studies during infection and determine whether bacteria also undergo similar morphological modifications under antibiotic treatment in vivo.

**Fig. 2:**
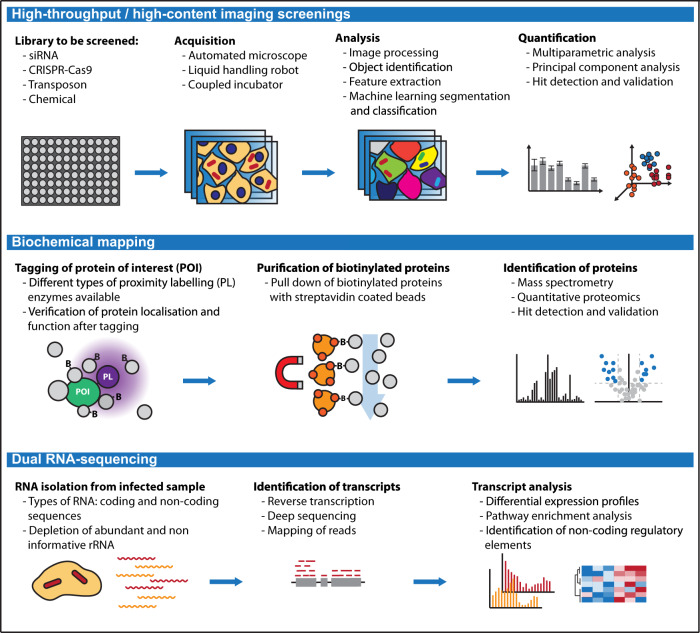
Scheme comparing advanced approaches to discover host and pathogen factors underlying infection. (Top) High-throughput microscopy usually screens for chemicals, host or bacterial factors that impact bacterial replication, dissemination or fitness. (Middle) Biochemical mapping, a proteomic screening approach to identify interaction partners of host and bacterial proteins of interest. (Bottom) Dual RNA-sequencing for the identification of differentially expressed transcripts from the host and pathogen.

Affinity purification followed by mass spectrometry is a traditional approach to identify interaction partners of host or bacterial factors. However, enzyme-catalysed proximity-labelling techniques (also known as biochemical mapping) can be used as a powerful alternative (Fig. 2 and Table 1B). Here, an enzyme producing a reactive biotin derivative is either targeted to a subcellular compartment or tagged to a bait protein, which results in biotinylation of neighbouring proteins inside the living cell. Due to the high affinity between biotin and streptavidin, the resulting biotinylated proteins can be purified and identified by mass spectrometry. Considering that biotinylation occurs promiscuously within a ~10 nm radius from the enzyme, it is not limited to direct interaction partners and also includes other proximal proteins. There are two main enzymes engineered for biochemical mapping: BioID (derived from a biotin ligase of *Escherichia coli*)^41^ and APEX (an ascorbate peroxidase from soybean)^42^. Proximity biotinylation has been used to investigate the composition and assembly of the T6SS in enteroaggregative *E. coli*^43^. Here, APEX2 was tagged to the T6SS key component TssA, which identified TagA, a protein that stops polymerisation of the T6SS sheath and limits its contraction. In addition, the authors expressed APEX2-TssA in various mutants blocked at different stages of T6SS assembly. This approach enabled the temporal dissection of protein interactions required for T6SS biogenesis. Other groups have explored the use of biochemical mapping during infection of tissue culture cells. For example, BioID was used to identify interaction partners of *S*. Typhimurium T3SS effectors^44^. Considering that introducing lengthy tags on secreted bacterial effectors frequently impairs their translocation across the bacterial membrane, the authors expressed tagged effectors directly in human epithelial cells. From this, they identified ~400 protein partners for the selected effectors, confirming previously known interactions and also discovering unknown ones, such as the biogenesis of lysosome-related organelle complex (BLOC)-2 as a host target of SifA, an important *Salmonella* effector required for the formation of *Salmonella* induced filaments (SIFs) at the BCV. In a study using *C. trachomatis*, authors used APEX2 to tag two inclusion membrane proteins (IncA and IncF) without impairing their secretion^45^. Inc proteins are T3SS translocated effectors that incorporate into inclusions bodies, the intracellular vacuole that sustains *C. trachomatis* replication. Since Inc proteins contain large hydrophobic regions that make their immunoprecipitation challenging, APEX2 tagging was efficient to identify their molecular partners, including LRRF1 (a host protein important for inclusion biogenesis and acquisition of host nutrients by bacteria). It is increasingly recognised that proximity-labelling techniques offer potential to map host–pathogen interactions, considering their success in multiple cell biology studies mapping the proteomic composition of subcellular regions (such as the mitochondria-ER contact sites^46^). A key advantage is that labelling occurs in live cells, which limits detection of non-specific interactions (e.g. proteins that belong to separate cellular compartments) normally detected by traditional pull downs. In addition, biochemical mapping has been useful to identify transient protein interactions (however, in this case, quantitative proteomics are generally required to avoid false positives). Recently, labelling enzymes have progressively been modified to achieve higher reaction efficiency and shorter labelling times (Bio-ID2^47^, TurboID^48^ and miniTurbo^48^). This, together with the use of fragment complementation for several enzymes, will ensure a broader application of biochemical mapping in cellular microbiology.

A separate method for the exploration of host–pathogen interactions includes the profiling of RNA expression patterns that occur during infection. In a typical transcriptomic experiment, RNA is isolated from the sample of interest, reverse transcribed to cDNA, sometimes amplified, sequenced and mapped to a genome of reference. Understanding RNA expression profiles of bacteria collected from infected samples compared to broth has provided unexpected insights on pathogenic adaptations to infection^49^. An important advance in this field has been the development of ‘dual RNA-seq’, which enables the simultaneous transcriptome analysis from both host and pathogen, thus capturing the reciprocal response between both organisms in a single experiment^49^ (Fig. 2 and Table 1B). One significant limitation that had to be overcome for the application of dual RNA-seq is based on intrinsic differences between RNA from prokaryotic and eukaryotic cells, which comprise variations in length, composition (e.g. polyA tail), processing (splicing) and their relative abundance in the infected sample (amount of RNA in a eukaryotic cell can be 100–200 times larger than in a prokaryotic cell). Several techniques have been developed to overcome this limitation, including enrichment of bacterial transcripts. Among these, it is useful to note the development of Pathogen Hybrid Capture (PatH-Cap)^50^, in which bacterial transcripts are isolated using specific biotinylated RNA probes pulled out using streptavidin beads. RNA-seq has proven valuable to understand the infection process for bacterial species which are poorly amenable to genetic manipulation, such as the obligate intracellular pathogen *Orientia tsutsugamushi*^51^. Interestingly, a comparison between two *Orientia* strains (Karp and UT 176) showed little overlap between their transcripts and differed substantially in the host cell inflammatory response. By integration of their transcriptional profiles with comparative genomics and proteomics, authors identified antisense RNA as a major mechanism of gene regulation in this neglected pathogen. Future dual RNA-seq studies will benefit from ongoing developments in sensitivity offered by third-generation sequencing techniques. In addition, further insights will be gained from the application of this technique to infected tissues (to understand the complexity underlying the infection process of different cell types) and to single-cells^52^ (to unravel heterogeneity that naturally occurs at the population level).

## Innovative infection models for cellular microbiology

**Fig. 3:**
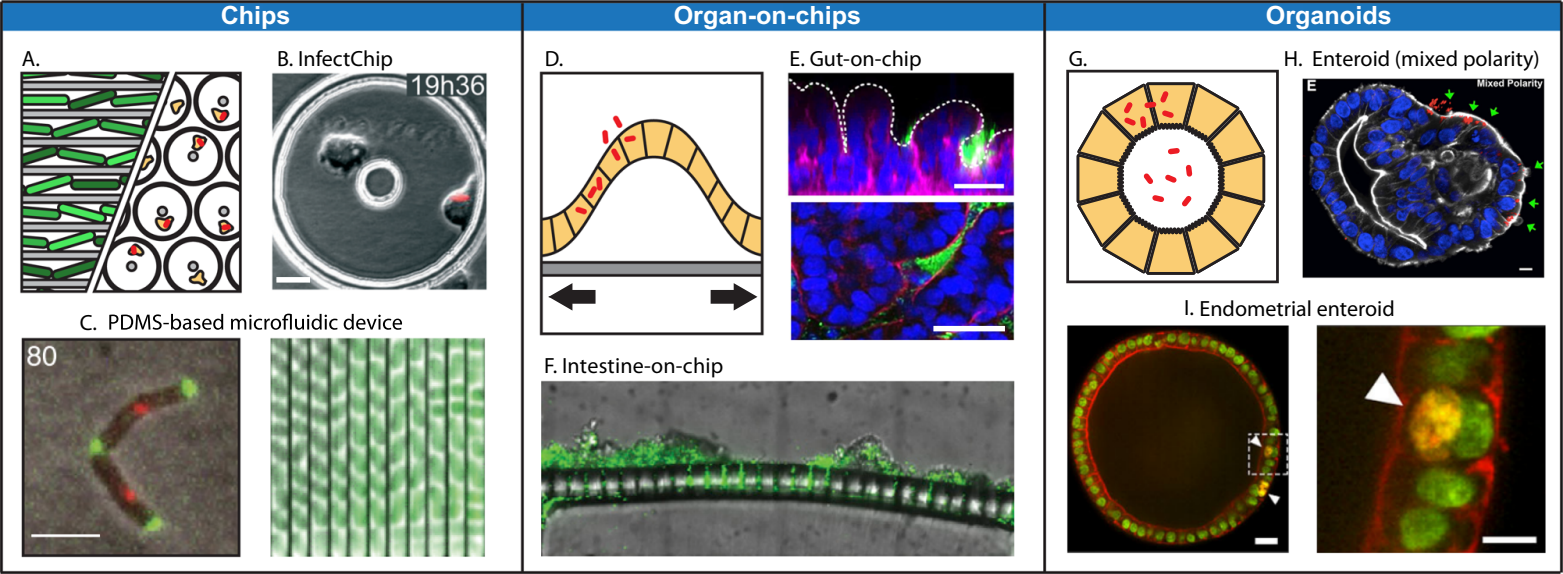
Innovative platforms (chips, organs-on-chips and organoids) recently used to dissect the infection process. **A** Schematic diagram of chips containing bacteria or infected cells. **B** InfectChip containing two cells of the *D. discoideum* species, one of them infected with mCherry expressing *M. marinum* (scale bar = 10 μm, modified from Delincé et al.^54^). **C** PDMS-based microfluidic devices for the single-cell analysis of bacteria. (Left) *M. smegmatis* expressing Wag31-GFP to label the bacterial septum and mCherry-DnaN to mark the DNA replisome complex, cultured in a microfluidic chip (scale bar = 3 μm, modified from Santi et al.^97^). (Right) *S*. Typhimurium expressing GFP under control of the promoter *sicA* to assess T3SS-1 activity under exposure of antibiotics grown on a microfluidic device over time (x axis) for single-cell analysis (modified from Arnoldini et al.^98^). **D** Schematic diagram of organ-on-chip infected with bacteria. **E** *E. coli* expressing GFP infecting epithelial cells on a gut-on-chip (upper panel, vertical cross-section; bottom panel horizontal cross-section, scale bar = 50 μm) showing F-actin in magenta and nuclei in blue (modified from Kim et al.^99^). **F** Frontal cross-section of an intestine-on-chip infected with *S. flexneri* expressing GFP (modified from Grassart et al.^56^). **G** Schematic diagram of organoids infected with bacteria. **H** *S*. Typhimurium expressing mCherry infecting the apical surface of an enteroid with mixed epithelial polarity (actin in white, nuclei in blue, scale bar = 10 μm, modified from Co et al.^61^). **I** (Left) Murine endometrial organoid infected with *C. trachomatis* expressing mCherry (actin in red, DNA i green, scale bar = 40 μm, modified from Bishop et al.^100^). (Right). Higher magnification of boxed area shown in J (scale bar = 20 μm).

The incorporation of ‘microfluidic chips’ into microscopy routines has revolutionised the analysis of single bacterial cells and their infection process (Fig. 3 and Table 1C). Generally, these devices entrap and isolate individual cells for prolonged visualisation over time using live-cell microscopy. This is particularly useful in the case of bacteria (which can be motile or subject to Brownian motion) and highly motile phagocytic cells, as they can escape from the field of view during long-term imaging experiments. Individual cell tracking offers the detection and precise characterisation of minor phenotypes often masked within the heterogeneity of any given population. These types of studies have been essential to understand the mechanisms underlying the persistence of *M. smegmatis* to isoniazid, an antitubercular antibiotic^53^. Due to the disparity in size between bacterial pathogens (~1 μm) and host cells (~50 μm), the design of microfluidic devices for their co-culture has been a challenge. A successful design includes InfectChip, a microfluidic device developed for long-term single-cell time-lapse microscopy of bacterial cell—amoeba interactions, that can be potentially adapted for other host cells, including macrophages^54^. InfectChip has been used to dissect the diverse outcomes of *Klebsiella pneumoniae* and *M. marinum* infection of the soil amoeba *Dictyostelium discoideum*^54,55^. This approach has been shown to effectively monitor and quantify early and late events during host–pathogen interplay at the single-cell level. These events, difficult to analyse using standard microscopy techniques at the bulk population level, were revealed to occur in an asynchronous and heterogeneous manner. In addition, microfluidic devices allow timely control of the perfusion medium, which can be modified during the course of the infection experiment to study the impact of starvation or antibiotics.

Different microfluidic chips have been developed to mimic the environment of infected human organs in a controlled manner (Fig. 3). This is the case of Intestine-Chip, used to assess the early stages of infection by *S. flexneri*^56^. Here, human colonic epithelial cells proliferate and develop into villi- and crypt-like structures on a semipermeable membrane coated with an extracellular matrix. This 3D arrangement of the epithelium was instrumental for T3SS expression and efficient invasion of *S. flexneri*, which increased ~10,000-fold (as compared to other conventional 2D cell support systems). In addition, the application of mechanical forces (to recreate peristalsis) in Intestine-Chip significantly improved this invasion step. These results challenge a long-standing dogma for *S. flexneri* invasion: *S. flexneri* were viewed to invade ‘M cells’ of the intestinal ‘Peyer’s patches’ and subsequently spread basolaterally through the enteric epithelium. Although this dogma could explain the low invasion efficiency of *S. flexneri* in traditional cell culture systems, these results using Intestine-Chip show that recapitulation of 3D characteristics and mechanical forces of the invaded tissue are essential for the early steps of the infection process. Another microfluidic device, called Lung-on-Chip, has been used to recreate the microenvironment of pulmonary alveoli during infection with *M. tuberculosis*^57^. In this microfluidic device, a porous membrane supports the growth of a mixture of endothelial cells found in lung alveoli and GFP expressing bone marrow-derived macrophages. Here, the authors found that defective surfactant production by alveolar epithelial cells resulted in faster intracellular growth of *M. tuberculosis*. The addition of exogenous surfactant partially removed virulence-associated lipids from the *M. tuberculosis* cell surface, and impaired their growth in macrophages, highlighting the role of pulmonary surfactant for innate immune defence against tuberculosis. Taken together, organ-on-chip strategies can enable the isolated investigation of key processes underlying infection of complex tissues and organs, difficult to perform in vivo using animal models.

Considering their biomedical relevance, ‘organoids’ are promising infection models to study bacterial pathogenesis, as they mimic in vivo characteristics of human organs while still amenable to experimental manipulation (Fig. 3 and Table 1C). The type of organoids derived from human intestinal stem cells or induced pluripotent stem cells are called enteroids, and are highly suited to study intestinal infection. Studies using enteric organoids include infection by intestinal pathogens *Helicobacter pylori*^58^, *S*. Typhimurium^59^ and several pathotypes of *E. coli*^60^. In all cases, bacteria are microinjected into the lumen of spherical enteroids, where they invade enterocytes and colonise the epithelium. Injecting bacteria in individual organoids is typically performed manually, and reduces the throughput potential of the experiment. However, recently developed apical-out enteroids revert this polarity, and the apical side of the epithelium is exposed to the external medium. Using this approach, infection with *S*. Typhimurium occurs upon the addition of bacteria to the medium^61^. While most studies using enteroids have focused on the integrity of the epithelial barrier during infection, the future of organoids has great potential. Organoids are amenable to several genetic manipulation techniques and represent an exciting platform for drug screens. In addition, human-derived organoids can enable the investigation of commensal and pathogenic bacteria that are restricted to humans (and currently lack an animal model for their study). Considering that organoids can be derived from human biopsies that maintain a patient’s genetic background, it is tempting to speculate that organoids will be key for the future development of personalised treatments.

Taken together, chips, organ-on-chips and organoids represent promising platforms to assess complex infection phenotypes in a highly controlled manner, despite some limitations (Fig. 3 and Table 1C). Chips are valuable to analyse phenotypes at the single-cell level, but one limitation is that chips are specifically designed for (or at least adapted to) the host and pathogen in question, which restricts their broad use. In contrast, several organ-on-chips are commercially available (e.g. for intestine and lung) and useful to study infection by a wide range of pathogens. Organ-on-chips are ideal to explore the influence of physical properties and mechanical forces on the infected epithelium but are limited in recapitulating the complexity of the whole organ response. Organoids more closely reproduce the physiology of the organ they mimic. However, organoids are significantly more challenging to image at high resolution, and they are also less amenable to high-throughput studies. The development of standardised protocols and increased accessibility will likely expand the number of cellular microbiology labs that routinely include these platforms to study the infection process.

## Use of in vivo infection models for human health impact

The host response to infectious disease is complex, influenced by multiple factors, including virulence of the pathogen and host genetic make-up. Here we highlight four widely used animal models to study cellular microbiology*: Caenorhabditis elegans* (worm), *Drosophila melanogaster* (fruit fly), *Danio rerio* (zebrafish) and *Mus musculus* (mouse), which have significantly contributed to our understanding of the infection process in vivo (Box 2).

Due to its ease of genetic manipulation, rapid generation time, simple body plan and transparency, the nematode *C. elegans* is an animal model that has provided fundamental biological discovery, such as the mechanism of RNA silencing. In recent years, *C. elegans* has emerged as a powerful invertebrate model to study infection biology. Lacking phagocytic cells such as macrophages, *C. elegans* is useful to study highly conserved pathways of immunity in intestinal epithelial cells. A key strength of the system is the straightforward infection protocol: bacteria are provided as a food source. The opportunistic bacterial pathogen *Pseudomonas aeruginosa* causes a lethal intestinal infection in *C. elegans*, and is the first and best-studied bacterial pathogen in this model. *P. aeruginosa* is an extracellular pathogen in *C. elegans*, but endocytosis of the bacterial-produced Exotoxin A can upregulate transcription factors controlling host defence genes (such as CEBP-2, an ortholog of the mammalian C/EBPγ important in infection and inflammation)^62^. Although other bacterial pathogens can infect *C. elegans*, including *S. flexneri*, *L. monocytogenes* and *L. pneumophila*, only *S*. Typhimurium can cause intracellular infection (reviewed in Balla and Troemel^63^). Significantly, *C. elegans* has been informative to study the role of biofilm formation^64^ and microbiota^65^ in vivo. *Enterococcus faecium* is a human commensal bacterium used as a probiotic in livestock, yet its mechanism of protection against enteric infection was unknown. In a recent study using *C. elegans*, *E. faecium* was shown to secrete a peptidoglycan hydrolase that produces peptidoglycan fragments improving host ‘tolerance’ to infection by *S*. Typhimurium^66^. This study suggested that enzymes (such as *E. faecium* peptidoglycan hydrolases) from commensal bacteria can be used to enhance probiotic treatments.

A widespread invertebrate model for infection is *D. melanogaster*. Infectious disease research using *Drosophila* is highlighted by the discovery of the innate immune receptor Toll and subsequent identification of homologous Toll-like receptors (TLRs) in humans and mice. TLRs recognise conserved molecules present in microbes, called pathogen-associated molecular patterns (PAMPS), and lead to activation of innate immune signalling pathways. The strength of *Drosophila* as an infection model is due to its conserved innate immune pathways with mammals, small size and fast generation time, as well as the availability of genetic manipulation tools (making it amenable to screening campaigns). *Drosophila* has been used to perform genetic screens at the whole organism level to identify factors underlying host resistance to *L. monocytogenes* infection^67^. Surprisingly, whole organism results failed to significantly overlap with results from S2 *Drosophila* derived cells^33^, showing the importance of doing screens at both the single-cell (studying cell-autonomous immunity) and whole organism level (studying the complex interplay between immune cells and infected tissues)^68^. *Drosophila* has also been used to model gastrointestinal infection^69^ and the role of commensal microbiota in host defence^70^. Finally, *Drosophila* is an important model to study the interplay between metabolism and innate immune responses. As one example, cytokines have been shown to dysregulate the autophagy-mediated accumulation of triglycerides in *Drosophila* macrophages, which promotes *M. marinum* survival^71^.

Zebrafish larvae have transformed our understanding of host–pathogen interactions because of their fully annotated genome (highly homologous to that of humans), genetic manipulability and translational potential^72^. In addition, their optical transparency is exceptional for non-invasive in vivo microscopy of innate immune development and host–pathogen interactions. The imaging potential of zebrafish larvae has recently been showcased by the development of LLSM in combination with adaptive optics. This provides subcellular imaging with an extraordinary spatiotemporal resolution to visualise multiple biological processes in vivo, including organelle dynamics or neural circuit development^73^. Infection of zebrafish larvae with *M. marinum* (a fish pathogen) is recognised as a natural host–pathogen system to model mycobacterial infection in humans. Zebrafish infection is often used to investigate roles for bacterial autophagy in vivo^74,75^, and has recently elucidated roles for the pro-inflammatory cytokine tumour necrosis factor (TNF) during necrosis of infected macrophages^76^. In this case, the intracellular pathway that mediates a TNF response involves multiple interconnected organelles including the mitochondria, lysosome and ER^77^. Interestingly, this discovery identified several druggable targets with therapeutic potential (such as ER ryanodine receptors). The repertoire of pathogens studied using the zebrafish model has significantly expanded to now include a wide variety of bacterial pathogens, including *Mycobacterium leprae*, *P. aeruginosa* and *V. cholerae*, underscoring their versatility and discovery potential (reviewed in Gomes and Mostowy^72^). Infection in the hindbrain ventricle has been useful to study intra- and inter-species bacterial interactions, such as competition and predation, inside the host. Signals to induce the *Staphylococcus aureus* T7SS are poorly understood, yet its activity during zebrafish infection can be exploited to test the function of T7SS effectors in vivo, including TspA shown to depolarise the membrane of competitor bacteria^78^. In a separate study, predatory bacteria *Bdellovibrio bacteriovorus* were shown to cooperate with the host innate immune system to clear antimicrobial-resistant *S. flexneri*, which may inspire the use of predatory bacteria as an alternative to antibiotics to treat bacterial infection in humans^79^.

Mice have been the premiere animal model to study infectious diseases for many years, and are necessary for preclinical trials to test the dose and efficacy of treatments and vaccines. Production of knockout mice in inbred strains (including C57BL/6 or BALB/c) are typically used to demonstrate the in vivo relevance of host defence mechanisms discovered previously in tissue culture cells or non-vertebrate animal models. In the case of *Shigella*, a neuronal apoptosis inhibitory protein (NAIP)- NLR family CARD domain-containing protein 4 (NLRC4) ‘inflammasome’ deficient mouse model has been generated, which is susceptible to *S. flexneri* and recapitulates the clinical characteristics of bacillary dysentery in humans^80^. Importantly, these results suggest that the lack of inflammasome response in human epithelial cells is a major reason for the efficient colonisation of the human intestine by *S. flexneri*. However, results using mice do not always recapitulate results using tissue culture cells. As a consequence, alternative animal models are being explored. One example is the use of infant rabbits to study *S. flexneri* infection, which reproduces the bloody diarrhoea phenotype and serves to dissect processes of bacterial invasion and dissemination in vivo^81^. Another example in which the mouse model did not reflect results obtained in vitro is that of bacterial autophagy, where a study in ATG5 deficient mice showed increased susceptibility to *M. tuberculosis* infection independent of canonical autophagic activity^82^.

To understand the role of microbiota in gut colonisation by bacterial pathogens, a variety of mouse models for studying infection resistance to *S*. Typhimurium (which causes a typhoid-like disease in mice) have been proposed^83^. Commensal *Bacteroides* species have been shown to produce the short fatty acid propionate, which disrupts intracellular pH and prevents *S*. Typhimurium proliferation^84^. A major limitation of the mouse model is their natural resistance to some enteropathogenic bacteria, yet humanised mice have been a successful strategy to increase the repertoire of pathogens studied in vivo. For infection by *S. enterica* serovar Typhi (which is normally restricted to humans), immunocompromised mice can be grafted with human immune cells to support bacterial proliferation^85^. In the case of *L. monocytogenes* infection, a key innovation was the development of transgenic mice expressing human E-cadherin in enterocytes^86^. Human E-cadherin (which differs from mouse E-cadherin at amino acid position 16) can interact with the *L. monocytogenes* invasion protein InlA, thus promoting bacterial internalisation. These humanised mice are susceptible to *Listeria* infection, and were transformative to study bacterial invasion of the intestinal epithelium^86^ and placental barrier^87^. Recent studies have shown that species-specific to the mouse microbiota (such as Clostridiales) significantly promote resistance to colonisation by *L. monocytogenes*^88^. While targeting specific bacterial competitors (present in the mouse microbiome, absent in the human microbiome) may generate more relevant mouse models to study *L. monocytogenes* infection, their protective potential when administered as probiotics in humans remains to be studied. Alternatively, transient depletion of mouse microbiota may favour intestinal exposure to engineered *L. monocytogenes* carrying a highly immunogenic antigen^89^. This approach to boost the immune response may be a useful platform to study vaccination at the intestinal mucosa in humans.

Despite their limitations to fully capture the physiopathology of human infections, animal models remain a cornerstone in cellular microbiology to study the complexity of host–pathogen interactions. While only a fraction of human pathogens has been studied using animal models, their increasing application to study unknown bacterial species will accelerate our ability to combat emerging and neglected infectious diseases.

Box 2 Modelling bacterial infection at the whole animal level

*Caenorhabditis elegans* (worm): *C. elegans* is a transparent unsegmented nematode first established as a model organism by Sidney Brenner to study neural development. The use of *C. elegans* has enabled fundamental advance significantly contributing to multiple aspects of biology. This is highlighted by the Nobel Prize-winning discovery of short interference RNA (siRNA), which inspired targeted protein depletion and genome-wide screens in a wide range of tissue culture cells and animal models. For infection biology, *C. elegans* has been used to study antimicrobial peptides and the lack of professional phagocytic cells makes worms convenient to investigate gut epithelial cell immunity. *C. elegans* is also used for innovative transgenerational studies, investigating mechanisms underlying inheritance of behaviour and pathogen avoidance.

*Drosophila melanogaster* (fruit fly): *D. melanogaster* is a non-vertebrate model organism first used by Thomas Hunt Morgan to study genetic inheritance. Research using *D. melanogaster* has been awarded several Nobel Prizes, including one for research on the Toll signalling pathway which motivated the characterisation of Toll-like receptors (TLRs) as sensors of innate immunity in humans and other animals. Since then, *D. melanogaster* is widely used to study host factors involved in infection (due to its genetic tractability and the wide variety of mutants available) and is mostly focused on antimicrobial peptides, humoral immunity and macrophage-like haemocytes (*D. melanogaster* primary immune cells). Recent studies are modelling infection in the fly gut epithelium to study the role of the microbiota, diet and metabolism in host defence.

*Danio rerio* (zebrafish): pioneered by George Streisinger to study vertebrate development, *D. rerio* is an important animal model for biomedical research. The genetic tractability of zebrafish and its amenability to non-invasive high-resolution microscopy makes *D. rerio* ideal to investigate host–pathogen interactions from the single cell to the whole animal level. Considering that zebrafish larvae lack an adaptive immune response, studies are often performed to understand the role of the innate immune system (in particular macrophages and neutrophils) in host defence. Zebrafish are emerging as a versatile system to study cell-autonomous immunity (pathogen recognition, autophagy), bacteria–bacteria interactions (competition, predation) and to discover unforeseen mechanisms of infection control.

*Mus musculus* (mouse): *M. musculus* is a small rodent and (since its first use in the 17th century by William Harvey) has become the mostly used mammalian model in biomedical research because of its close proximity to human physiology. In vivo research using mice is widely performed due to the large community of inbred and genetically modified transgenic lines available, useful to map or study host factors underlying resistance/susceptibility to infection. Moreover, a variety of ‘humanised’ models have been developed to render mice susceptible to human-specific pathogens. Infection of mice is clinically relevant and routine before advancing clinical trials testing pharmacologic treatments on humans.

## Summary and perspectives

A more complete understanding of host–pathogen interactions is required to improve therapeutics for human health. Here, we overview innovative technologies transforming the field of cellular microbiology, enabling fundamental discovery in both infection and cell biology. However, the field faces important challenges to influence global health in an efficient manner (Box 3, outstanding questions). First, the crisis of antimicrobial resistance will require a concerted international effort to deliver alternative treatments to fight infections. This will involve creative strategies such as searching for antibiotics among commensal bacteria from other non-human organisms^90^, predicting molecules with an antibacterial activity using machine learning^91^ and/or promoting treatments to precisely modulate the patient’s immune response. Regarding antimicrobials, more studies are needed to address the impact of clinically-approved drugs on the microbiota to provide a more tailored administration that benefits the individual patient. While *Listeria*, *Shigella* and *Salmonella* are recognised as paradigms for cellular microbiology research, increasing the repertoire of bacterial pathogens under investigation will be important to discover unexpected pathogenic mechanisms required for host cell infection. The development of tools for the investigation of genetically intractable organisms, including the obligate intracellular bacteria *Rickettsia*^92^ and *Orientia*^51^, is rendering poorly understood pathogens more amenable to cellular microbiology research. Considering the adaptation of bacterial strains to the lab, increasing the study of clinical isolates for cellular microbiology will be invaluable to capture the full breadth of pathogenic agents and mechanisms of disease circulating in the human population. In terms of the host, the study of human genetic immunodeficiencies that predispose to infection (i.e. the human model) in combination with cellular microbiology has great potential to directly impact public health. As one example, inborn genetic errors in type-I-IFN immunity has been shown to predispose to severe COVID-19 disease^93,94^. Finally, increasing the technical capacity and throughput of manipulating patient cells and tissues in vitro can help to address the heterogeneity underlying human susceptibility to infection, and promote the exploration of personalised treatments.

Box 3 Outstanding questions

- Considering the rapid evolution of microscopy techniques, the resolution will improve and unforeseen insights will emerge. What innovations in microscopy and other technologies can help to revolutionise future cellular microbiology?
- The availability of innovative infection platforms (such as chips, organs-on-chips and organoids) and animal models have transformed approaches to study cellular microbiology. For an improved understanding of pathogenesis and fundamental cellular processes, how faithfully do these platforms/animal models have to model natural human infection?
- We are only beginning to understand bacteria–bacteria interactions and the role of complex bacterial secretion systems. In this context, what is the influence of infection (and subsequent antibiotic treatment) on microbiota, community architecture and whole animal health?
- *Listeria*, *Shigella* and *Salmonella* are paradigms of cellular microbiology research. How can the investigation of poorly understood bacterial species and clinical strains expand our understanding of the infected cell?
- For over 30 years, cellular microbiology has provided extensive insights on mechanisms of bacterial pathogenesis and host response. How will advances in the field shape future medicine and more broadly impact human health?

## References

[1] Stradal TEB, Schelhaas M (2018). Actin dynamics in host–pathogen interaction. FEBS Lett..

[2] Welch MD, Iwamatsu A, Mitchison TJ (1997). Actin polymerization is induced by Arp2/3 protein complex at the surface of Listeria monocytogenes. Nature.

[3] Schermelleh L (2019). Super-resolution microscopy demystified. Nat. Cell Biol..

[4] Wang L, Frei MS, Salim A, Johnsson K (2019). Small-molecule fluorescent probes for live-cell super-resolution microscopy. J. Am. Chem. Soc..

[5] Singh MK, Kenney LJ (2021). Super-resolution imaging of bacterial pathogens and visualization of their secreted effectors. FEMS Microbiol. Rev..

[6] Huang F (2016). Ultra-high resolution 3D imaging of whole cells. Cell.

[7] Zhang Y, Lara-Tejero M, Bewersdorf J, Galán JE (2017). Visualization and characterization of individual type III protein secretion machines in live bacteria. Proc. Natl Acad. Sci. USA.

[8] Thurston TLM (2012). Galectin 8 targets damaged vesicles for autophagy to defend cells against bacterial invasion. Nature.

[9] Perrin AJ, Jiang X, Birmingham CL, So NSY, Brumell JH (2004). Recognition of bacteria in the cytosol of mammalian cells by the ubiquitin system. Curr. Biol..

[10] Otten EG (2021). Ubiquitylation of lipopolysaccharide by RNF213 during bacterial infection. Nature.

[11] Cemma M, Kim PK, Brumell JH (2011). The ubiquitin-binding adaptor proteins p62/ SQSTM1 and NDP52 are recruited independently to bacteria-associated microdomains to target *Salmonella* to the autophagy pathway. Autophagy.

[12] Van Wijk SJL (2017). Linear ubiquitination of cytosolic Salmonella Typhimurium activates NF-κB and restricts bacterial proliferation. Nat. Microbiol..

[13] Chen B-C (2014). Lattice light-sheet microscopy: Imaging molecules to embryos at high spatiotemporal resolution. Science.

[14] Moore AS (2021). Actin cables and comet tails organize mitochondrial networks in mitosis. Nature.

[15] Wassie AT, Zhao Y, Boyden ES (2019). Expansion microscopy: principles and uses in biological research. Nat. Methods.

[16] Zwettler FU (2020). Molecular resolution imaging by post-labeling expansion single-molecule localization microscopy (Ex-SMLM). Nat. Commun..

[17] Lim Y (2019). Mechanically resolved imaging of bacteria using expansion microscopy. PLoS Biol..

[18] Dewachter L, Fauvart M, Michiels J (2019). Bacterial heterogeneity and antibiotic survival: understanding and combatting persistence and heteroresistance. Mol. Cell.

[19] Walter T (2017). Incorporation studies of clickable ceramides in Jurkat cell plasma membranes. Chem. Commun..

[20] Götz R (2020). Nanoscale imaging of bacterial infections by sphingolipid expansion microscopy. Nat. Commun..

[21] Weiner A, Enninga J (2019). The pathogen–host interface in three dimensions: correlative FIB/SEM applications. Trends Microbiol..

[22] López-Jiménez AT (2018). The ESCRT and autophagy machineries cooperate to repair ESX-1-dependent damage at the Mycobacterium-containing vacuole but have opposite impact on containing the infection. PLoS Pathog..

[23] Bernard EM (2020). *M. tuberculosis* infection of human iPSC-derived macrophages reveals complex membrane dynamics during xenophagy evasion. J. Cell Sci..

[24] Weiner A (2016). Macropinosomes are key players in early *Shigella* invasion and vacuolar escape in epithelial cells. PLoS Pathog..

[25] Kühn S (2020). Actin assembly around the *Shigella*-containing vacuole promotes successful infection. Cell Rep..

[26] Sedzicki, J. et al. 3D correlative electron microscopy reveals continuity of *Brucella*-containing vacuoles with the endoplasmic reticulum. *J. Cell Sci*. **131**, jcs210799 (2018). 10.1242/jcs.210799 29361547

[27] Kreshuk A, Zhang C (2019). Machine learning: advanced image segmentation using ilastik. Methods Mol. Biol..

[28] Oikonomou CM, Jensen GJ (2019). Electron cryotomography of bacterial secretion systems. Microbiol. Spectr..

[29] Fattinger SA (2020). *Salmonella* Typhimurium discreet-invasion of the murine gut absorptive epithelium. PLoS Pathog..

[30] Lobato-Marquez D (2021). Mechanistic insight into bacterial entrapment by septin cage reconstitution. Nat. Commun..

[31] Jiang H (2014). Stable isotope imaging of biological samples with high resolution secondary ion mass spectrometry and complementary techniques. Methods.

[32] Greenwood DJ (2019). Subcellular antibiotic visualization reveals a dynamic drug reservoir in infected macrophages. Science.

[33] Agaisse H (2005). Genome-wide RNAi screen for host factors required for intracellular bacterial infection. Science.

[34] Philips JA, Rubin EJ, Perrimon N (2005). Drosophila RNAi screen reveals CD36 family member required for mycobacterial infection. Science.

[35] Jones TR (2009). Scoring diverse cellular morphologies in image-based screens with iterative feedback and machine learning. Proc. Natl Acad. Sci. USA.

[36] Berg S (2019). ilastik: interactive machine learning for (bio)image analysis. Nat. Methods.

[37] Stringer C, Wang T, Michaelos M, Pachitariu M (2020). Cellpose: a generalist algorithm for cellular segmentation. Nat. Methods.

[38] Fisch D (2019). Defining host–pathogen interactions employing an artificial intelligence workflow. Elife.

[39] Brodin P, Christophe T (2011). High-content screening in infectious diseases. Curr. Opin. Chem. Biol..

[40] Peters JM (2016). A comprehensive, CRISPR-based functional analysis of essential genes in bacteria. Cell.

[41] Roux KJ, Kim DI, Raida M, Burke B (2012). A promiscuous biotin ligase fusion protein identifies proximal and interacting proteins in mammalian cells. J. Cell Biol..

[42] Martell JD (2012). Engineered ascorbate peroxidase as a genetically encoded reporter for electron microscopy. Nat. Biotechnol..

[43] Santin YG (2018). In vivo TssA proximity labelling during type VI secretion biogenesis reveals TagA as a protein that stops and holds the sheath. Nat. Microbiol..

[44] D’Costa VM (2019). BioID screen of Salmonella type 3 secreted effectors reveals host factors involved in vacuole positioning and stability during infection. Nat. Microbiol..

[45] Olson, M. G. et al. Proximity labeling to map host-pathogen interactions at the membrane of a bacterium-containing vacuole in chlamydia trachomatis-infected human cells. *Infect. Immun*. **87**, e00537-19 (2019). 10.1128/IAI.00537-19 PMC6803327 31405957

[46] Han Y (2019). Directed evolution of split APEX2 peroxidase. ACS Chem. Biol..

[47] Kim DI (2016). An improved smaller biotin ligase for BioID proximity labeling. Mol. Biol. Cell.

[48] Branon TC (2018). Efficient proximity labeling in living cells and organisms with TurboID. Nat. Biotechnol..

[49] Westermann AJ, Vogel J (2021). Cross-species RNA-seq for deciphering host–microbe interactions. Nat. Rev. Genet..

[50] Betin V (2019). Hybridization-based capture of pathogen mRNA enables paired host-pathogen transcriptional analysis. Sci. Rep..

[51] Mika-Gospodorz B (2020). Dual RNA-seq of *Orientia tsutsugamushi* informs on host-pathogen interactions for this neglected intracellular human pathogen. Nat. Commun..

[52] Penaranda C, Hung DT (2019). Single-cell RNA sequencing to understand host-pathogen interactions. ACS Infect. Dis..

[53] Wakamoto Y (2013). Dynamic persistence of antibiotic-stressed mycobacteria. Science.

[54] Delincé MJ (2016). A microfluidic cell-trapping device for single-cell tracking of host–microbe interactions. Lab. Chip.

[55] López-Jiménez, A. T., Hagedorn, M., Delincé, M., McKinney, J. & Soldati, T. The developmental cycle of *Dictyostelium discoideum* ensures curing of a mycobacterial infection at both cell-autonomous level and by collaborative exclusion. Preprint at *bioRxiv*10.1101/586263 (2019).

[56] Grassart A (2019). A bioengineered human organ-on-chip reveals that mechanical forces and 3D topology of the intestinal epithelium are critical for *Shigella* infection. Cell Host Microbe.

[57] Thacker VV (2020). A lung-on-chip model of early *M. tuberculosis* infection reveals an essential role for alveolar epithelial cells in controlling bacterial growth. Elife.

[58] Schlaermann P (2016). A novel human gastric primary cell culture system for modelling *Helicobacter pylori* infection in vitro. Gut.

[59] Zhang Y-G, Wu S, Xia Y, Sun J (2014). *Salmonella*-infected crypt-derived intestinal organoid culture system for host-bacterial interactions. Physiol. Rep..

[60] Rajan, A. et al. Novel segment- and host-specific patterns of enteroaggregative *Escherichia coli* adherence to human intestinal enteroids. *MBio* **9**, e02419-17 (2018). 10.1128/mBio.02419-17 PMC5821088 29463660

[61] Co JY (2019). Controlling epithelial polarity: a human enteroid model for host-pathogen Interactions. Cell Rep..

[62] Reddy KC, Dunbar TL, Nargund AM, Haynes CM, Troemel ER (2016). The *C. elegans* CCAAT-enhancer-binding protein gamma is required for surveillance immunity. Cell Rep..

[63] Balla KM, Troemel ER (2013). *Caenorhabditis elegans* as a model for intracellular pathogen infection. Cell. Microbiol..

[64] Desai SK, Padmanabhan A, Harshe S, Zaidel-Bar R, Kenney LJ (2019). *Salmonella* biofilms program innate immunity for persistence in *Caenorhabditis elegans*. Proc. Natl Acad. Sci. USA.

[65] Zhang J, Holdorf AD, Walhout AJ (2017). *C. elegans* and its bacterial diet as a model for systems-level understanding of host–microbiota interactions. Curr. Opin. Biotechnol..

[66] Rangan KJ (2016). A secreted bacterial peptidoglycan hydrolase enhances tolerance to enteric pathogens. Science.

[67] Ayres JS, Freitag N, Schneider DS (2008). Identification of *Drosophila* mutants altering defense of and endurance to *Listeria monocytogenes* infection. Genetics.

[68] Clatworthy AE, Romano KP, Hung DT (2018). Whole-organism phenotypic screening for anti-infectives promoting host health perspective. Nat. Chem. Biol..

[69] Lee KA (2018). Inflammation-modulated metabolic reprogramming is required for DUOX-dependent gut immunity in *Drosophila*. Cell Host Microbe.

[70] Douglas AE (2018). The *Drosophila* model for microbiome research. Lab. Anim..

[71] Peán CB (2017). Regulation of phagocyte triglyceride by a STAT-ATG2 pathway controls mycobacterial infection. Nat. Commun..

[72] Gomes MC, Mostowy S (2020). The case for modeling human infection in zebrafish. Trends Microbiol..

[73] Liu, T. L. et al. Observing the cell in its native state: imaging subcellular dynamics in multicellular organisms. *Science* **360**, eaaq1392 (2018). 10.1126/science.aaq1392 PMC6040645 29674564

[74] Mostowy S (2013). The zebrafish as a new model for the in vivo study of *Shigella flexneri* interaction with phagocytes and bacterial autophagy. PLoS Pathog..

[75] Van Der Vaart M (2014). The DNA damage-regulated autophagy modulator DRAM1 links mycobacterial recognition via TLP-MYD88 to authophagic defense. Cell Host Microbe.

[76] Roca FJ, Ramakrishnan L (2013). TNF dually mediates resistance and susceptibility to mycobacteria via mitochondrial reactive oxygen species. Cell.

[77] Roca FJ, Whitworth LJ, Redmond S, Jones AA, Ramakrishnan L (2019). TNF induces pathogenic programmed macrophage necrosis in tuberculosis through a mitochondrial-lysosomal-endoplasmic reticulum circuit. Cell.

[78] Ulhuq FR (2020). A membrane-depolarizing toxin substrate of the *Staphylococcus aureus* type VII secretion system mediates intraspecies competition. Proc. Natl Acad. Sci. USA.

[79] Willis AR (2016). Injections of predatory bacteria work alongside host immune cells to treat *Shigella* infection in zebrafish larvae. Curr. Biol..

[80] Mitchell PS (2020). NAIP–NLRC4-deficient mice are susceptible to shigellosis. Elife.

[81] Yum LK, Byndloss MX, Feldman SH, Agaisse H (2019). Critical role of bacterial dissemination in an infant rabbit model of bacillary dysentery. Nat. Commun..

[82] Kimmey JM (2015). Unique role for ATG5 in neutrophil-mediated immunopathology during *M. tuberculosis* infection. Nature.

[83] Kreuzer M, Hardt WD (2020). How food affects colonization resistance against enteropathogenic bacteria. Annu. Rev. Microbiol..

[84] Jacobson A (2018). A gut commensal-produced metabolite mediates colonization resistance to *Salmonella* Infection. Cell Host Microbe.

[85] Song J (2010). A mouse model for the human pathogen *Salmonella* Typhi. Cell Host Microbe.

[86] Lecuit M (2001). A transgenic model for listeriosis: Role of internalin in crossing the intestinal barrier. Science.

[87] Disson O (2008). Conjugated action of two species-specific invasion proteins for fetoplacental listeriosis. Nature.

[88] Becattini S (2017). Commensal microbes provide first line defense against *Listeria monocytogenes* infection. J. Exp. Med..

[89] Becattini S (2020). Enhancing mucosal immunity by transient microbiota depletion. Nat. Commun..

[90] Imai Y (2019). A new antibiotic selectively kills Gram-negative pathogens. Nature.

[91] Stokes JM (2020). A deep learning approach to antibiotic discovery. Cell.

[92] Burke TP (2020). Inflammasome-mediated antagonism of type I interferon enhances *Rickettsia* pathogenesis. Nat. Microbiol..

[93] Zhang Q (2020). Inborn errors of type I IFN immunity in patients with life-threatening COVID-19. Science.

[94] Pairo-Castineira E (2020). Genetic mechanisms of critical illness in Covid-19. Nature.

[95] Mostowy S (2010). Entrapment of intracytosolic bacteria by septin cage-like structures. Cell Host Microbe.

[96] Basler M, Pilhofer M, Henderson GP, Jensen GJ, Mekalanos JJ (2012). Type VI secretion requires a dynamic contractile phage tail-like structure. Nature.

[97] Santi I, Dhar N, Bousbaine D, Wakamoto Y, McKinney JD (2013). Single-cell dynamics of the chromosome replication and cell division cycles in mycobacteria. Nat. Commun..

[98] Arnoldini M (2014). Bistable expression of virulence genes in *Salmonella* leads to the formation of an antibiotic-tolerant subpopulation. PLoS Biol..

[99] Kim HJ, Li H, Collins JJ, Ingber DE (2016). Contributions of microbiome and mechanical deformation to intestinal bacterial overgrowth and inflammation in a human gut-on-a-chip. Proc. Natl Acad. Sci. USA.

[100] Bishop RC, Boretto M, Rutkowski MR, Vankelecom H, Derré I (2020). Murine endometrial organoids to model *Chlamydia* infection. Front. Cell. Infect. Microbiol..

